# Identification of Two Novel Mutations in KDM3A Regulatory Gene in Iranian Infertile Males

**DOI:** 10.29252/.23.3.220

**Published:** 2019-05

**Authors:** Zohreh Hojati, Elaheh Soleimanpour, Seyed-Morteza Javadirad, Mohammad Hossein Nasr-Esfahani

**Affiliations:** 1 Department of Biology, Faculty of Sciences, University of Isfahan, Isfahan, P. O. Box 81746-73441, Iran; 2 Isfahan Fertility and Infertility Center, Isfahan, Iran; 3 Andrology and Embryology Department, Reproductive Medicine Research Center and Cell Sciences Research Center Royan Institute, (Isfahan Campus), ACECR, Tehran, Iran

**Keywords:** KDM3A, Male infertility, Spermatogenic failure

## Abstract

**Background:**

*KDM3A* is a key epigenetic regulator expressed in the testis and is required for packaging and condensation of sperm chromatin. To this point, the association of the *KDM3A* gene with infertility has not been studied in human. The aim of this study was to screen any new mutation in *KDM3A* gene to explore more details of human male infertility.

**Methods:**

In this work, 150 infertile men (oligozoospermia and azoospermia) and 150 normal healthy fathers were studied. Polymerase chain reaction-single-strand conformation polymorphism (PCR-SSCP) and sequencing were used to screen any mutation in exons 12, 22, and 24 of *KDM3A*.

**Results:**

The infertile men showed various SSCP patterns for the exons 12 and 24, but not for exon 22. A transversion point mutation in exon 12 and a single nucleotide deletion in exon 24 were detected using sequencing analysis. The transversion mutation was located in the preceding exon of lysine-specific demethylase1 and Jumonji (Jmj)-C domain and the later one (deletion) in the cupin-like motif of KDM3A protein. Neither Y chromosome microdeletions nor partial azoospermia factor deletion was found in these patients.

**Conclusion:**

The mutations found in infertile men with otherwise unexplained severe spermatogenic failure could be considered as the origin of their abnormalities.

## INTRODUCTION

The couple’s reproductive failure after two years of unprotected sexual intercourse are called infertility, and male side defects account for 50% of infertility[1,2]. Two-thirds of infertile men have sperm production difficulties, including undescended testis, infections, heat, sperm antibodies, varicocele, drugs, or radiation damages[3]. Mammalian spermatogenesis is a unique and also a complex process and involves physiological, biochemical and morphological changes[4]. During spermatogenesis, histone replacement is occurred via histone-to-protamine transformation[5]. On the other hand, histone tails can post-translationally be modified to promote the activation or the repression of their underlying genes[6]. Histones methylation and demethylation, especially for histone three (H3), are essential steps of successful spermatogenesis[7-9]. The most studied sites of histone tails already modified are lysines 4, 9, 27, 36, and 79 of H3 and lysine 20 of H4[10,11]. In contrast, lysine-specific demethylase1 and Jumonji (Jmj)-C domain containing proteins are involved in the elimination of the methyl group of histones and could activate or silence the genes[12].

The Jmj-C domain containing proteins are composed of 44 proteins and grouped into four clusters; lysine-specific demethylase 3 A (*KDM3A*) is a member of cluster 2[13,14]. *KDM3A* (NG_047167.1) was first recognized in a testis c-DNA library and revealed that this protein is highly expressed in male germ cell[15]. *KDM3A* null mice showed up to 90% reduction in *TNP1* mRNA level when compared with wild types; therefore, *TNP1* is necessary for sperm maturation[16,17].

According to the catalogue of somatic mutations in cancer (COSMIC v85 database), missense substitution (69.49%), synonymous substitution (22.06%), and nonsense substitution (9.93%) are the most prevalent mutations detected for *KDM3A*[18]. All the 169 point-mutations recorded in Cosmic v85 database are completely based on germ cell tumors, and no mutation has been documented for normal testis tissue. In this work, for the first time, the existence of *KDM3A* gene mutation was investigated in non-cancerous testis tissue of human infertile men to reveal any relationship between this gene and infertility.

## MATERIALS AND METHODS

### Patients

The study was approved by the University of Isfahan, Isfahan, Iran. We obtained written informed consents from all patients, family members, and control subjects who participated in the study. Normal healthy fathers (n = 150) have participated voluntarily in this work. Semen and blood samples from 150 infertile men were collected by Isfahan Fertility and Infertility Center following the institutional review board approval of Royan Institute (Tehran, Iran) and Isfahan Fertility and Infertility Center. The age of the infertile men ranged from 23 to 65 years. Detailed information of their life style, such as smoking, alcohol and drug use, exposure to physical or chemical agents during their work or life, surgical history, and family history were recorded in a standard questionnaire.

### Semen analysis

Semen samples were analyzed according to WHO criteria. A number of factors were normally determined during semen analysis including semen volume (a measure of how much semen a man produces), sperm count (the number of sperms per ml of semen in one ejaculation), sperm motility (the percentage of sperms moving normally), sperm morphology (the percentage of sperms with a normal shape, tail, and head), and white blood cell count, though there were no blood cells in the semen. These men were then categorized into two groups: oligozoospermia and azoospermia.

### DNA extraction

Genomic DNA was extracted from peripheral blood samples using salting-out protocol[19]. To detect mutations, we used polymerase chain reaction-single-strand conformation polymorphism (PCR-SSCP) in conjunction with sequencing.

### PCR amplification

Three sets of specific primers were designed using Oligo®5 for screening any mutation that might exist in each of the exons 12, 22, and 24. PCR was performed using 20 ng of template DNA, 250 pmol of each dNTP, 5 pmol of each primer, 25 pmol of MgCl_2_, and 1U of *Taq* DNA polymerase in a final volume of 25 ml. For exon 13, the PCR reactions were carried out for 32 cycles (denaturation at 94 °C for 1 min, annealing at 64 °C for 1 min, and extension at 72 °C for 1 min) and finally 10 min at 72 °C. An initial denaturation of 4 min at 94 °C was also considered for both exons. For exon 22, denaturation was at 94 °C for 1 min, annealing at 68.8 °C for 45 seconds, and extension at 72 °C for 1 min and finally at 72 °C for 10 min. For exon 25, included denaturation was at 94 °C for 1 min, annealing at 58.4 °C for 1 min, and extension at 72 °C for 1 min and finally at 72 °C for 10 min. An initial denaturation of 4 min at 94 °C was also considered for all exons.

Different sequence-tagged site (STS) primers were used in this study; their sequences are available from GenBank under the accession numbers: sY1161, G66148, sY1191, G73809, sY1291, G72340, sY1206, G68331, and sY1201. These primers were employed in three different multiplex-PCRs. The PCR conditions were almost similar to those used for the amplification of the exon 22, except for the annealing temperatures.

### SSCP and sequencing analysis

Due to a very high accuracy in the determination of any mutation, SSCP was selected for mutation screening in the desired region of *KDM3A* gene[20,21]. Polyacrylamide gel electrophoresis was applied for analyzing the PCR products. Two separate stages were considered in this method, denaturing of PCR products and the electrophoresis of denatured products on non-denaturing polyacrylamide gel with neutral pH. A volume of 10 µl of PCR product was mixed in a SSCP buffer (98% formamide, 10 mM of EDTA, 0.05% bromophenol blue, and 0.05% xylene cyanol), with 2:1 ratio, kept at 95 °C for 10 min and immediately incubated on ice for 10 min. Optimal electrophoresis conditions were achieved after performing various optimizations. The gel was used at a concentration of 10% (29 ml of milli-Q H_2_O, 2.5 ml of Tris-aceta- EDTA 10×, 5 ml of glycerol, 12.5 ml of 40% Acrylamide/Bis-acrylamide, 300 µl of 10% ammonium persulfate, and 32 µl of Tetramethylethylenediamine. The gel was then silver-stained and visualized.

Samples with different SSCP patterns were PCR-amplified and sequenced (Sinaclone Company Tehran, Iran). The data were then analyzed using NCBI-BLAST, a search tool that compares the sequenced variants with NCBI nucleotide collection database and is based on GRCh38.p12 Primary Assembly.

## RESULTS

### SSCP analysis

In this work, 150 infertile men, including 60 azoospermic and 90 oligozoospermia with non-obstructive etiology, were studied. Specific PCR primers were designed to target exons 12, 22, and 24 (Table 1).

**Table 1:** Different primers that were used for mutation screening in exons 13, 22 and 25 *KDM3A* gene

Exon	Name	Primer sequence	PCR product (bp)
12	13-F	5’-GCTTCCCCTAGGTTACAATTCAACAAACAT-3’	225
	13-R	5’-GTGTGGCTCAATAGTTGACATAGCTTCCTT-3’	
22	22-F	5’-GGCAGCCTTTGTTGTCTTTTGTAAAACT-3’	176
	22-R	5’-ACCCTACCTTCTTCTTGCTCACACTGTC-3’	
24	25-F	5’-TCTAGGTATCAGAAGAGCAAGGTCAA-3’	159
	25-R	5’-TAAACACCACATCCCCAAGAAA-3’	

**Fig. 1:**
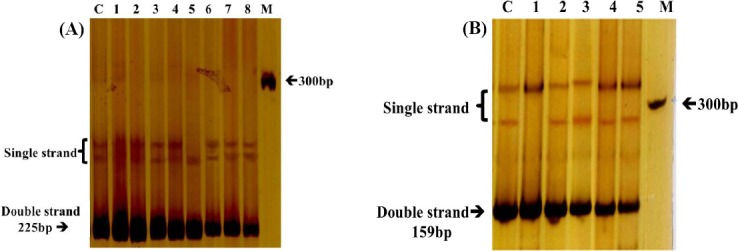
The study of mutations in exons 12 (A) and 24 (B) of *KDM3A* by SSCP. Lane C, healthy fertile person; lane M, DNA marker (100 bp); For A, lanes 1-4 and 6-8, infertile patients with no mutation; lane 5, infertile patient with mutation. For B, lane 1, the infertile patient with mutation, and lines 2-5 infertile patients with no mutation.

All the PCR reactions and SSCP analysis were carried out in duplicate to assure the reproducibility of the results. All the observed SSCP patterns of exon 12 were similar in almost all the PCR products that had been obtained from different infertile patients and the control group. Two infertile patients showed altered SSCP patterns for this exon; the patterns were unique and obviously vary with the others (Fig. 1A). No mobility shifts in patters were seen among all the patients and controls. Altered SSCP patterns of exon 24 were also observed among three infertile men, in comparison with other infertile and normal control individuals (Fig. 1B).

### Sequencing analysis

**Fig. 2:**
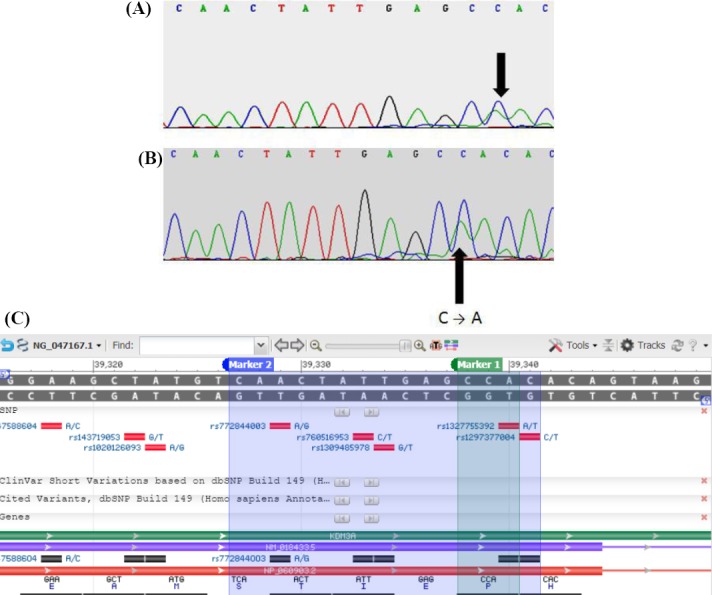
Schematic representation of the mutated exon 12 according to the sequencing and NCBI gene graphics. Exon 12 point mutation transversion C39339→A related to proline 645 to glutamine substitution has been shown by sequencing analysis in two patients (A and B). Panel C illustrates the reference nucleotide from NCBI Reference Sequence Database. The arrows show the point of mutation according to sequence results.

**Fig. 3:**
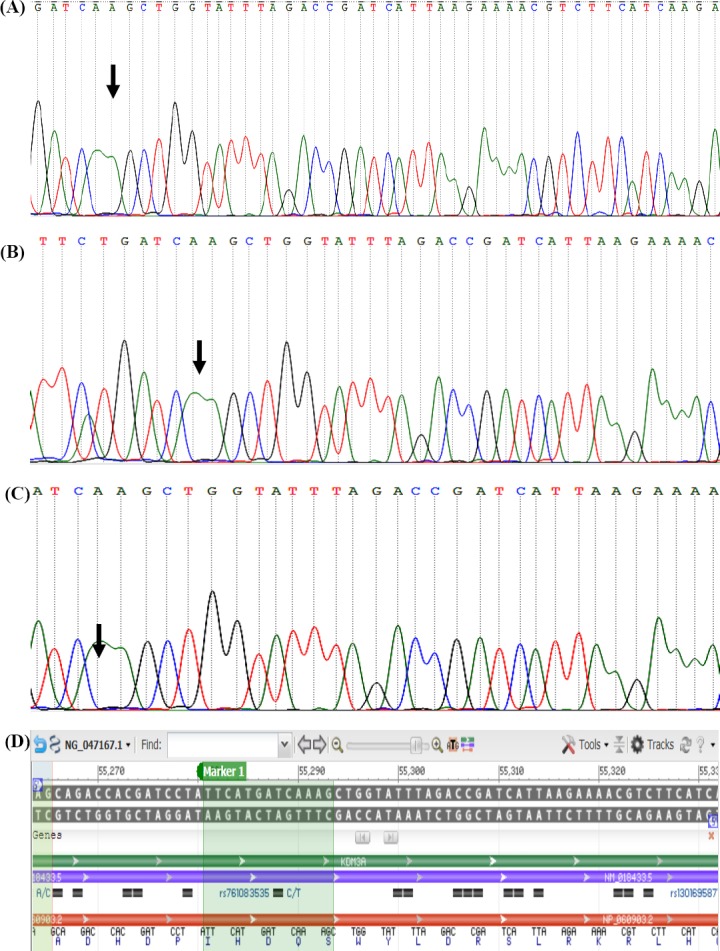
A single adenine nucleotide deletion at position 3956, related to amino acid at position 1211 in three infertile patients in exon 24 (A-C). Panel D illustrates the reference nucleotide from NCBI Reference Sequence Database. The arrows show the point of A-nucleotide deletion mutation according to sequence results.

**Fig. 4:**
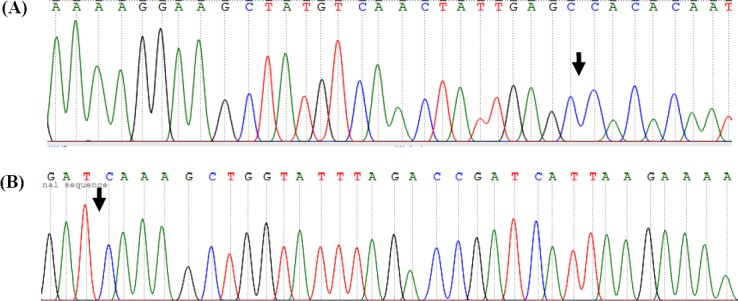
Sequencing results of exon 12 (A) and exon 24 (B) using DNA of control individuals. The result indicated no differences with the NCBI, NM_018433.5 equivalent to Homo sapiens KDM3A. The arrows show the interest point of mutation according to sequence results.

Sequencing analysis coupled with BLAST-type homology search against human *KDM3A* gene (NG_047167.1) demonstrated a point mutation transversion C39339→A, related to amino acid at position 645 (NP_060903.2, proline to glutamine) in the exon 12 of two patients (Fig. 2). A single nucleotide (A) deletion was also detected at position 55290, which was associated with glutamine at Position 1210 in the exon 24 in three infertile patients (Fig. 3). Since single nucleotide deletion changes the frame of the DNA and its coded protein, it can disturb the function and operation of the KDM3A protein. One of these patients had an infertile brother, and this matter can reveal the important effects of this gene on infertility. Therefore, by further mutation screening on this gene and infertile family of these patients, it may be regarded as a new gene marker for men’s infertility. DNA samples from fertile men were also subjected to sequencing as a control group (Fig. 4). BLAST analysis revealed 100% homology with *KDM3A* reference sequence NM_018433.5 indexed by NCBI (data not shown).

### Y chromosome microdeletions and partial azoospermia factors (AZF) deletions

**Fig. 5:**
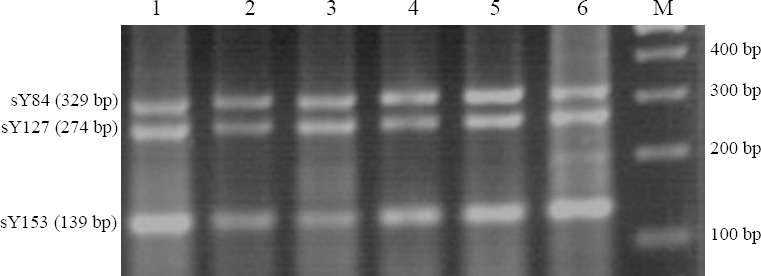
The results of multiplex PCR containing three markers sY153, sY127, and sY84. Lane 1, a normal healthy father (volunteer); lanes 2-6, different patients with mutation in their *KDM3A* gene; lane M, marker 100 bp.

Individuals who did carry a mutation in the exon 12 (two patients) and 24 (three patients) were studied in more details for the presence of Yq microdeletions by multiplex PCR (Fig. 5). Each of these five patients was separately tested using eight markers from the AZF loci by the following STS primers: AZFa (sY84), AZFb (sY87 and sY127), AZFc (sY254 and sY255 sY153), As illustrated in Figure 5, no microdeletion was observed in these patients. The patients were also screened for partial AZFc deletions by another multiplex PCR using another set of STS primers: sY1291, sY1191, sY1201, and sY1161. Besides, one single PCR reaction, containing (STS) primer sY1206, was used to screen the partial deletion in the AZFc region. Finally, neither Y chromosome microdeletion nor partial AZF deletion was found in these five patients. The data from this part has partially been shown here (Fig. 5).

## DISCUSSION

A few single-gene defects that cause male infertility have been identified in humans, but the function and performance of the *KDM3A* gene in humans is still unclear. Thus, there is uncertainty over the role of *KDM3A* gene in the incidence of infertility in humans. A previous study on Loss-of-function mutation has indicated the necessity of KDM3A for mouse spermatogenesis[17]. Okada and co-workers[17] introduced a large deletion downstream of exon 10 to block the coding region of functional Jmj-C domain. The exon 10 downstream displayed that *KDM3A* regulates the expression of its specific target genes such as transition nuclear proteins and protamine. They also established that the deletion of *KDM3A* could lead to oligozoospermia and finally infertility. Further studies on *KDM3A* exons 21 and 22 have shown a decreased expression level of *ACT* (cAMP-response element modulator activator) in the testes of *KDM3A* null mice, and consequently, the expression levels of all *ACT* target genes, including *Tnp1*, *Tnp2*, *Prm1*, and *Prm2*, significantly reduced[16].

The results from our study demonstrated 91% similarity between mice and human *KDM3A*, using Clustal W1.83 software (data not shown here). As *KDM3A* is likely important for human fertility, introduction of any mutation in this gene could lead to infertility. Consequently, the existence of any mutation in *KDM3A* gene investigated in infertile men (azoospermia and oligozoospermia with more than 90% head abnormality) using PCR-SSCP. Oligozoospermic men were selected for the present investigation as *KDM3A* regulates the genes responsible for sperm condensation.

Bioinformatics investigations have revealed that human *KDM3A* mRNA (NM_018433.5) contains 26 exons, and the catalytic Jmj-C domain covers a region composing of exons 21 and 22. Since Jmj-C is considered as the functional domain, any mutation or deficiency in this domain or preceding exons might have impacts on this region and might lead to failure in proper protein function. In order to examine this hypothesis, we selected exon 22, as the first composer of Jmj-C, for screening any mutation that might influence on this functional domain. Exons 12 and 24 were also chosen as candidates of preceding and following exons regarded to Jmj-C domain. In this study, we observed a point mutation (C33838→A) in exon 12 in two patients; both of them were oligozoospermia. This mutation results in a proline to glutamine (CAA) substitution. Our results also showed that both of the patients have only one copy of mutated allele, indicating a heterozygote situation (CA genotype). Furthermore, another mutation was detected in three infertile patients, two patients with azoospermia and one with oligozoospermia showing 94% sperm head abnormality. This mutation was a single nucleotide deletion (A nucleotide) at position 3956, related to amino acid at position 1211. Since the single nucleotide deletions are considered as a frame shift mutations, a very significant change in protein structure and function can be created by these types of mutations. On the other hand, this mutation was located after Jmj-C catalytic domain of *KDM3A*. It could be interesting if the mentioned mutation could make an impact on sperm maturation and condensation.

As various Y chromosome microdeletions and partial AZF deletions are the main causes of male infertility, they were considered and studied here[22-27]. The five patients under study were subjected to further analysis by different multiplex PCR procedures. Here, SSCP was initially conducted on all patients. Then the possibility of Y chromosome microdeletions and partial AZF deletions were investigated. In this case, the presence of any mutation even in infertile patients who possess Y chromosome microdeletions and/or partial AZF deletion was also determined. Interestingly, neither Y chromosome microdeletion nor partial AZF deletion was found in these five patients. One of these patients has an infertile brother. In a similar work, Bashamboo *et al*.[28] sequenced NR5A1 in 315 men with idiopathic spermatogenic failure in order to test the hypothesis that mutations in NR5A1 gene cause male infertility. They identified missense mutations in seven men with severe spermatogenic failure, but they did not observe these mutations in the entire coding sequence of 359 normospermic men and 370 fertile male controls.

Here, we demonstrated that heterozygous mutations in *KDM3A* are associated with severe spermatogenic failure in otherwise healthy men. In an analysis of 150 men seeking infertility treatment because of spermatogenic failure, we identified the same heterozygous missense mutations in two patients, and an identical deletion mutation in three patients, the former one in proceeding domain and the later one (deletion) in the area located after Jmj-C domain of the protein. Using *in silico* PolyPhen-2 software, we found that the P645Q missense mutation is predicted to be probably damaged with a score of 0.999 (sensitivity of 0.14 and specificity of 0.99). Each of the mutant proteins presumably fails to transactivate gonadal promoters optimally. These mutations may be the reason for infertility in these five patients. Since this gene contains many other exons, the existence of any mutation in the other part of this gene remains to be screened. Further molecular genetics studies are required to be carried out in order to practically confirm the association of the *KDM3A* gene with male infertility. Since, this gene is located on chromosome number 2; it could be transferred to all offspring’s gene pool, regardless of their gender. Therefore, it is recommended for infertile couples to investigate the existence of these mutations in *KDM3A* gene prior to intracytoplasmic sperm injection or other treatments, in order to prevent its transmission to successive generations.

## References

[1] Esteves SC (2013). A clinical appraisal of the genetic basis in unexplained male infertility. Journal of human reproductive sciences.

[2] Tvrda E, Agarwal A, Alkuhaimi N (2015). Male reproductive cancers and infertility:a mutual relationship. International journal of molecular sciences.

[3] Tabong PTN (2013). Understanding the social meaning of infertility and childbearing:a qualitative study of the perception of childbearing and childlessness in Northern Ghana. PLOS one.

[4] Punab M, Poolamets O, Paju P, Vihljajev V, Pomm K, Ladva R, Korrovits P, Laan M (2017). Causes of male infertility:a 9-year prospective monocentre study on 1737 patients with reduced total sperm counts. Human reproduction.

[5] Awe S, Renkawitz-Pohl R (2010). Histone H4 acetylation is essential to proceed from a histone- to a protamine-based chromatin structure in spermatid nuclei of *Drosophila melanogaster*. Systems biology in reproductive medicine.

[6] Fuks F (2005). DNA methylation and histone modifications:teaming up to silence genes. Current opinion in genetics and development.

[7] Colaco S, Modi D (2018). Genetics of the human Y chromosome and its association with male infertility. Reproductive biology and endocrinology.

[8] Gunes S, Arslan MA, Hekim GNT, Asci R (2016). The role of epigenetics in idiopathic male infertility. Journal of assisted reproduction and genetics.

[9] Cui X, Jing X, Wu X, Yan M, Li Q, Shen Y, Wang Z (2016). DNA methylation in spermatogenesis and male infertility. Experimental and therapeutic medicine.

[10] Martin C, Zhang Y (2005). The diverse functions of histone lysine methylation. Nature reviews molecular cell biology.

[11] Mihola O, Trachtulec Z, Vlcek C, Schimenti JC, Forejt J (2009). A mouse speciation gene encodes a meiotic histone H3 methyltransferase. Science.

[12] Metzger E, Wissmann M, Schüle R (2006). Histone demethylation and androgen-dependent transcription. Current opinion in genetics and development.

[13] Klose RJ, Kallin EM, Zhang Y (2006). JmjC-domain-containing proteins and histone demethylation. Nature reviews genetics.

[14] Takeuchi T, Watanabe Y, Takano-Shimizu T, Kondo S (2006). Roles of jumonji and jumonji family genes in chromatin regulation and development. Developmental dynamics.

[15] Höög C, Schalling M, Grunder-Brundell E, Daneholt B (1991). Analysis of a murine male germ cell-specific transcript that encodes a putative zinc finger protein. Molecular reproduction and development.

[16] Liu Z, Zhou S, Liao L, Chen X, Meistrich M, Xu J (2010). Jmjd1a demethylase-regulated histone modification is essential for cAMP-response element modulator-regulated gene expression and spermatogenesis. The journal of biological chemistry.

[17] Okada Y, Scott G, Ray MK, Mishina Y, Zhang Y (2007). Histone demethylase JHDM2A is critical for Tnp1 and Prm1 transcription and spermatogenesis. Nature.

[18] Forbes SA, Tang G, Bindal N, Bamford S, Dawson E, Cole C, Kok CY, Jia M, Ewing R, Menzies A, Teague JW, Stratton MR, Futreal PA (2010). COSMIC (the Catalogue of Somatic Mutations in Cancer):a resource to investigate acquired mutations in human cancer. Nucleic acids research.

[19] Miller SA, Dykes DD, Polesky HF (1988). A simple salting out procedure for extracting DNA from human nucleated cells. Nucleic acids research.

[20] Kakavas VK, Plageras P, Vlachos TA, Papaioannou A, Noulas VA (2008). PCR-SSCP:a method for the molecular analysis of genetic diseases. Molecular biotechnology.

[21] Gasser RB, Hu M, Chilton NB, Campbell BE, Jex AJ, Otranto D, Cafarchia C, Beveridge I, Zhu X (2006). Single-strand conformation polymorphism (SSCP) for the analysis of genetic variation. Nature protocols.

[22] Vogt PH, Edelmann A, Kirsch S, Henegariu O, Hirschmann P, Kiesewetter F, Köhn FM, Schill WB, Farah S, Ramos C, Hartmann M, Hartschuh W, Meschede D, Behre HM, Castel A, Nieschlag E, Weidner W, Gröne HJ, Jung A, Engel W, Haidl G (1996). Human Y chromosome azoospermia factors (AZF) mapped to different subregions in Yq11. Human molecular genetics.

[23] Hopps CV, Mielnik A, Goldstein M, Palermo GD, Rosenwaks Z, Schlegel PN (2003). Detection of sperm in men with Y chromosome microdeletions of the AZFa, AZFb and AZFc regions. Human reproduction.

[24] Simoni M, Bakker E, Krausz C (2004). EAA/EMQN best practice guidelines for molecular diagnosis of y-chromosomal microdeletions. State of the art 2004. International journal of andrology.

[25] Giachini C, Guarducci E, Longepied G, Degl'Innocenti S, Becherini L, Forti G, Mitchell M, Krausz C (2005). The gr/gr deletion(s):a new genetic test in male infertility?. Journal of medical genetics.

[26] Ravel C, Chantot-Bastaraud S, El Houate B, Mandelbaum J, Siffroi JP, McElreavey K (2006). GR/GR deletions within the azoospermia factor c region on the Y chromosome might not be associated with spermatogenic failure. Fertility and sterility.

[27] Wu B, Lu NX, Xia YK, Gu AH, Lu CC, Wang W, Song L, Wang SL, Shen HB, Wang XR (2007). A frequent Y chromosome b2/b3 subdeletion shows strong association with male infertility in Han-Chinese population. Human reproduction.

[28] Bashamboo A, Ferraz-de-Souza B, Lourenco D, Lin L, Sebire NJ, Montjean D, Bignon-Topalovic J, Mandelbaum J, Siffroi JP, Christin-Maitre S, Radhakrishna U, Rouba H, Ravel C, Seeler J, Achermann JC, McElreavey K (2010). Human male infertility associated with mutations in NR5A1 encoding steroidogenic factor 1. American journal of human genetics.

